# Clinical significance of quantitative bone SPECT/CT in the evaluation of hand and wrist pain in patients with rheumatic disease

**DOI:** 10.1038/s41598-021-03874-9

**Published:** 2022-01-10

**Authors:** Chae Hong Lim, Hyun-Sook Kim, Kyung-Ann Lee, JongSun Kim, Soo Bin Park

**Affiliations:** 1 Department of Nuclear Medicine, Soonchunhyang University Hospital Seoul, Soonchunhyang University College of Medicine, Seoul, Korea; 2 Department of Rheumatology, Soonchunhyang University Hospital Seoul, Soonchunhyang University College of Medicine, Seoul, Korea

**Keywords:** Diseases, Medical research, Rheumatology

## Abstract

We investigated the diagnostic value of the maximum standardized uptake value (SUV) at hand and wrist joints for differentiating rheumatic diseases via bone single-photon emission computed tomography (SPECT)/computed tomography (CT). A total of 84 patients manifesting hand and wrist pain (58 women; age, 49.8 ± 15.4 years) were finally diagnosed with rheumatoid arthritis (RA, n = 42), osteoarthritis (OA, n = 16), fibromyalgia (FM, n = 2), and other rheumatic diseases (n = 24). The SUV of each patient was measured in 32 joints including the distal interphalangeal (DIP), proximal interphalangeal (PIP), metacarpophalangeal (MCP), and wrist joints bilaterally. Differences in pain and SUVs between specific rheumatic diseases were assessed using the chi-squared test or one-way analysis of variance. Using the highest SUV (hSUV) in each patient, the diagnostic performance in differentiating specific diseases was evaluated by receiver operating characteristic (ROC) curve analysis. Pain symptoms were present in 886 (33.0%) sites in a total of 2688 joints. In four joint groups (DIP, PIP, MCP, and wrist), the SUVs of joints with pain were significantly higher than those of pain-free joints (all *P* < 0.001). Active joint sites with higher SUVs than the median value of each joint group were the most common in RA (55.1%). RA showed the greatest hSUV in the PIP (3.0 ± 2.4), MCP (3.5 ± 3.4), and wrist (3.3 ± 1.9) joint groups. FM was characterized by the lowest hSUV of all joint groups. In ROC curve analysis, the cumulative hSUV of the PIP, MCP, and wrist joint groups showed good performance for evaluating RA (area under the curve (AUC), 0.668; *P* = 0.005). The summation of the hSUVs at all joint groups had an excellent predictive performance for FM (AUC, 0.878; *P* < 0.001). Consequently, the arthritic activity of the hand and wrist joints based on SUV differed according to specific rheumatic diseases. Quantitative SPECT/CT may provide objective information related to arthritic activity for differentiating specific rheumatic diseases.

## Introduction

Bone scintigraphy imaging using Tc-99m-labeled bone tracers such as Tc-99m methylene diphosphonate (MDP) has been widely used to investigate active bone formation. It is highly sensitive but not always specific, for the assessment of musculoskeletal disease. Single-photon emission computed tomography (SPECT)/computed tomography (CT) is potentially more accurate than planar scintigraphy imaging, as it is based on anatomical information in the CT scan^1^. However, the visual assessment of SPECT/CT remains subjective because it is based on relative tracer uptake. In state-of-the-art SPECT/CT, quantitative measurements using the maximum standardized uptake value (SUV) have been reported to provide useful information due to greater objectivity in the interpretation^2,3^.

Hand or wrist pain is one of the most frequent symptoms and reason for admission in rheumatology. It may occasionally be difficult to identify the cause of pain because of the extensive differential diagnosis and similar clinical manifestations^4,5^. The clinical assessment of joint lesions based on pain characteristics facilitates the detection of possible rheumatic diseases^6,7^. However, pain perception is not necessarily related to inflammation or structural changes reflecting tissue damage because it is also affected by environmental and psychological factors^8,9^. In these cases, the maximum SUV of joints measured using quantitative SPECT/CT provides more objective information for arthritic activity related to peripheral joint damage, independently of pain symptoms. The severity of pain sensitization and arthritic activity can also differ according to various pathophysiological mechanisms of specific rheumatic diseases. This feature may be useful in narrowing the possible rheumatic conditions in patients who are initially classified with undifferentiated arthritis.

In this study, we compared joint SUVs and pain symptoms in patients with rheumatic disease manifesting as hand and wrist joint pain. In addition, we also evaluated whether the joint SUVs differed according to specific rheumatic diseases, and could assist in its differential diagnosis.

## Materials and methods

### Study population

We retrospectively reviewed the medical records of 87 consecutive patients who underwent bone SPECT/CT of the hand and wrist region requested by our rheumatologist between September 2016 and March 2020. The patients had hand or wrist pain as the chief complaint with a known or suspected rheumatic disease at the time of the test. The primary indications for bone SPECT/CT were to evaluate scintigraphic activity of the hands that reflect articular inflammation and/or increased bone turnover and to confirm a clinical diagnosis. Among the candidates, three patients who had other possible causes (trauma, infection, or bone metastasis) besides rheumatic conditions for hand or wrist pain were excluded. Consequently, 84 patients with hand or wrist pain related to rheumatic disease were finally included. This study was approved by the Institutional Review Board (IRB) of Soonchunhyang University Hospital (IRB No. 2020-04-020) with an exemption for obtaining written consent from the study subjects. All retrospective analyses involving human participants were in accordance with the ethical standards of the institutional and/or national research committee and the principles of the 1964 Declaration of Helsinki and its later amendments or comparable ethical standards.

### Acquisition of quantitative SPECT/CT

All imaging data were acquired using a Symbia Intevo16 hybrid SPECT/CT system (Siemens Healthineers, Erlangen, Germany) comprised of an integrated dual-head SPECT camera with a 16-slice helical CT scanner. In all patients, the field of view (FOV) covered both hand and wrists completely, and immobilization materials were placed between patient’s fingers to minimize motion of hand during SPECT/CT imaging (Supplementary Figure S1). Tc-99m MDP was injected at a dose of 740 MBq (20 mCi) and SPECT was performed 3 h post-injection. SPECT images were acquired under the following parameters: low-energy high-resolution collimator, 256 by 256 matrix size, step-and-shoot mode, and dual-head gamma cameras with 45 steps per detector (angle of 4°) at 22 s/step. Subsequent to SPECT acquisition, the CT images were generated at 110 kV and 80 mA using the Auto-mA mode with a 256 × 256 matrix and 1.2 pitch and reconstructed into 1.5-mm slice thicknesses. The SPECT images were reconstructed using the ordered subset conjugate gradient maximization (xSPECT Bone) algorithm allowing SUV quantification, with CT-based zone information (1 subset and 24 iterations; Gaussian filter of 15.0)^10^. The SPECT voxel counts based on accurate correction were also converted to activity concentrations (Bq/mL) using a system planar sensitivity correction factor measured with a ^57^Co source during reconstruction^11^. Finally, the SPECT and CT images were co-registered as fusion SPECT/CT images.

### Review of SPECT/CT images and analysis of SUV uptake

**Figure 1:**
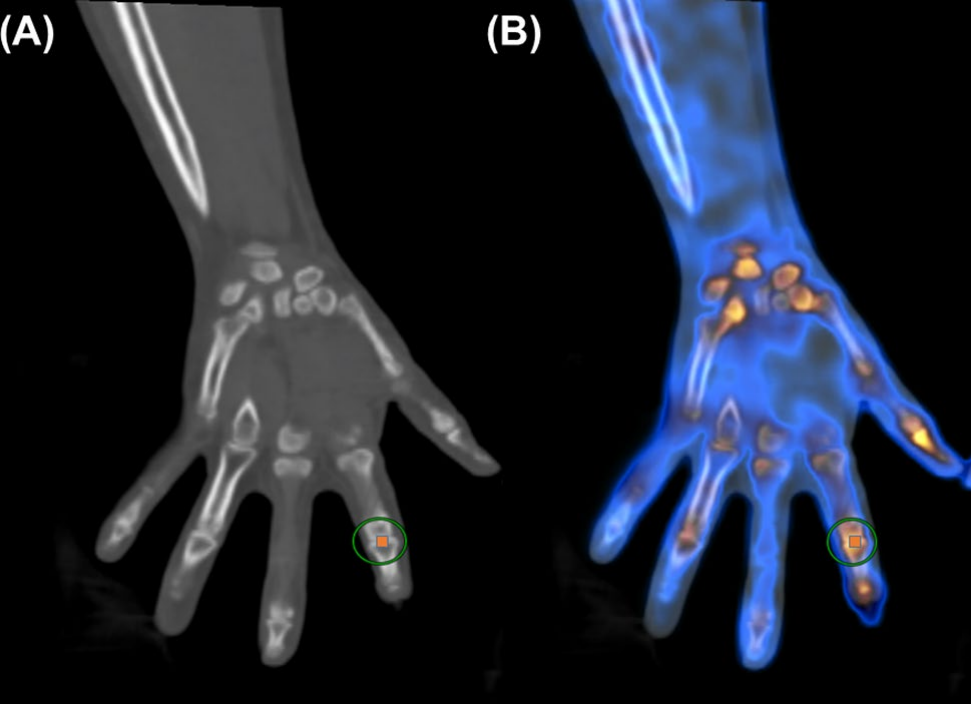
(**A**) CT and (**B**) SPECT/CT images demonstrate how the SUV was measured at the hand joint. The SUVmax was derived from a spherical VOI drawn over the periarticular region, placing the joint space at the center of the VOI.

SPECT/CT images were reviewed by a board-certified nuclear medicine physician using an image-analysis workstation (MIM version 6.6, MIM Software Inc., OH, USA). The joint uptake of each patient was quantified using the maximum SUV at a total of 32 joints in four joint levels including the distal interphalangeal (DIP) (n = 8), proximal interphalangeal (PIP) (n = 10), metacarpophalangeal (MCP) (n = 10), and wrist (n = 4). Using the CT images as an anatomic reference, a spherical volume of interest (VOI) was drawn over the periarticular region including the epiphysis, placing the joint space at the center of the VOI, which was automatically reflected on the SPECT/CT fusion images (Fig. 1). All joints of the total population (n = 2688) were divided into DIP, PIP, MCP and wrist joint groups according to the level of the joint. For lesion-based analysis, the active joint lesion was defined using the median value of SUVs (median SUV) in the population according to joint groups. In other word, the joint site with a value higher than the median SUV in the same joint group was considered an active joint lesion in this study. For patient group analysis, the highest SUV (hSUV) of each joint group in both hands was selected within each patient. Thus, each patient had four representative hSUVs, at DIP, PIP, MCP, and wrist joints.

### Clinical assessment

The final diagnosis of all subjects was made by the treating rheumatologist based on patient history, clinical examination, laboratory tests, and available imaging. All subjects were classified into four groups according to the prevalence and pathophysiology as rheumatoid arthritis (RA), osteoarthritis (OA), fibromyalgia (FM), and other rheumatic conditions. A pain localization diagram displaying the 32 joints of the hand and wrist was used to identify the pain location. The subjects marked the painful joint sites on the day they underwent SPECT/CT.

### Statistical analyses

Continuous variables were compared using the Student’s t-test (two variables) and analysis of variance (ANOVA) (more than two variables). Pearson’s chi-squared test was used for the comparison of categorical variables. Diagnostic performance of joint SUVs for predicting specific rheumatic diseases was assessed by the area under the curve (AUC) of the receiver operating characteristics (ROC) analysis. All statistical tests were two-sided with a significance level set at 0.05 and were performed via the Statistical Package for the Social Sciences version 23.0 (IBM Corp., Armonk, NY, USA) and MedCalc version 15.5 (MedCalc, Mariakerke, Belgium) software programs.

## Results

### Clinical characteristics

The baseline demographic and clinical characteristics of the 84 study subjects are summarized in Table 1. The study involved 26 men and 58 women, ranging in age from 18 to 86 years (mean ± SD, 49.8 ± 15.4 years). The most frequent rheumatic disease was RA (n = 42, 50.0%), followed by OA (n = 16, 19.0%). Two patients were diagnosed with FM. The remaining 24 cases were categorized as other rheumatic diseases, which included progressive systemic sclerosis (n = 5, 6.0%), palindromic rheumatism (n = 5, 6.0%), enteropathic arthritis (n = 3, 3.6%), psoriatic arthritis, and gout. The symptom of pain was present in 886 (33.0%) sites of a total of 2688 joints. The PIP joint was the most common joint site associated with pain (40.0%), followed by wrist (35.1%), MCP (29.9%), and DIP joints (26.9%).

**Table 1:** Clinical characteristics.

Variables	Number
Age (y), mean ± SD	49.8 + 15.4
Male gender	26/84 (31.0%)
**Disease classification**	
Rheumatoid arthritis	42/84 (50.0%)
Osteoarthritis	16/84 (19.0%)
Fibromyalgia	2/84 (2.4%)
Other rheumatic diseases	24/84 (28.6%)
**Joints with pain**	
DIP	181/672 (26.9%)
PIP	336/840 (40.0%)
MCP	251/840 (29.9%)
Wrist	118/336 (35.1%)

SD, standard deviation; DIP, distal interphalangeal; PIP, proximal interphalangeal; MCP, metacarpophalangeal.

### Quantitative assessment of hand and wrist joints

**Figure 2:**
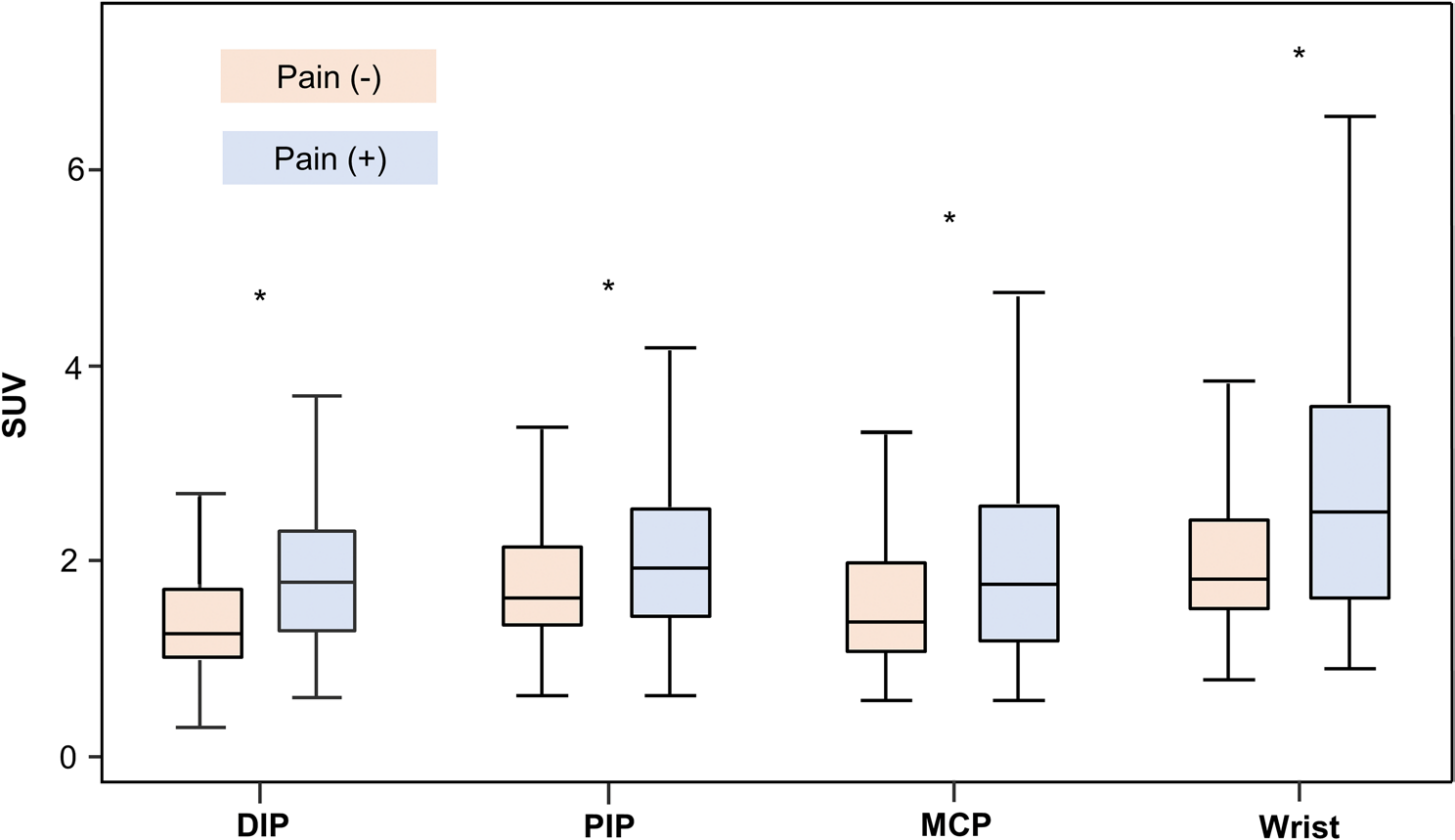
Box-whisker plots of joint SUV at each joint level. DIP, distal interphalangeal; PIP, proximal interphalangeal, MCP, metacarpophalangeal; *P < 0.05.

Six-hundred seventy DIP, 840 PIP, 840 MCP, and 336 wrist joints were analyzed in 84 patients. The mean values of SUVs gradually increased from distal to proximal in all joints: 1.5 ± 0.7 (median, 1.4) in DIP, 1.8 ± 1.0 (median, 1.6) in PIP, 1.8 ± 1.1 (median, 1.5) in MCP, and 2.3 ± 1.5 (median, 2.0) in the wrist joints. In all of four joint groups, the SUVs of joints with pain were significantly higher than those of pain-free joints (Fig. 2, all *P* < 0.001). A total of 1254 joints (46.7%) were classified as active joint lesions based on the median SUV at each joint group.

### Lesion-based analysis according to specific rheumatic conditions

Among the rheumatic conditions, joint pain occurred most frequently in OA (40.6%), followed by other rheumatic diseases (35.0%), RA (29.8%), and FM (12.5%). However, the rheumatic disease with the most active joint lesions was RA (55.1%), followed by other rheumatic diseases (42.1%), OA (36.5%), and FM (4.7%). Although there were fewer painful joint lesions (33.0%) than active joint lesions (46.7%) in the entire joint sites, it was the opposite in OA and FM (OA, 40.6% vs. 36.5%; FM, 12.5% vs. 4.7%; Table 2).

**Table 2:** Lesion-based analysis according to specific rheumatic conditions.

	RA (n = 1344)	OA (n = 512)	Other (n = 768)	FM (n = 64)	Total (N = 2688)	P value
**Present pain**						< 0.001
Painful joint site	29.8% (401)	40.6% (208)	35.0% (269)	12.5% (8)	33.0% (886)	
Pain-free joint site	70.2% (943)	59.4% (304)	65.0% (499)	87.5% (56)	67.0% (1802)	
**SUV based arthritic activity**						< 0.001
Active joint site^a^	55.1% (741)	36.5% (187)	42.1% (323)	4.7% (3)	46.7% (1254)	
Non-active joint site	44.9% (603)	63.5% (325)	57.9% (445)	95.3% (61)	53.3% (1434)	

^a^Active joint site is defined as an SUV > the median value at the same joint level. RA, rheumatoid arthritis; OA, osteoarthritis; Other, other rheumatic diseases; FM, fibromyalgia.

### Patient-based analysis according to specific rheumatic conditions

The hSUVs of each joint group were used to compare the disease activity according to specific rheumatic conditions in patient group (Table 3). When the hSUVs of each patient were compared regardless of the joint group, they were significantly different according to specific diseases (*P* = 0.009), and RA had the highest arthritic activity of joint lesion (3.0 ± 2.4). According to individual joint groups, the PIP (3.0 ± 2.4, *P* = 0.49), MCP (3.5 ± 3.4, *P* = 0.18), and wrist joints (3.3 ± 1.9, *P* = 0.31) showed higher hSUVs in RA than in non-RA diseases, but did not reach statistical significance. FM showed the least arthritic activity among all joint groups.

**Table 3:** Patient-based analysis according to specific rheumatic conditions.

	RA (n = 42)	OA (n = 16)	Other (n = 24)	FM (n = 2)	Total (N = 84)	P value
**All joint levels**						0.009
hSUV	3.0 ± 2.4	2.4 ± 1.7	2.3 ± 1.4	1.4 ± 0.2	2.6 ± 2.0	
**Individual joint level**						
hSUV at DIP	2.1 ± 1.1	2.1 ± 1.1	1.7 ± 0.5	1.3 ± 0.3	1.9 + 0.9	0.186
hSUV at PIP	3.0 ± 2.4	2.7 ± 2.0	2.3 ± 1.3	1.6 ± 0.2	2.7 ± 2.1	0.495
hSUV at MCP	3.5 ± 3.4	2.5 ± 2.0	2.2 ± 1.0	1.2 ± 0.0	2.9 ± 2.7	0.181
hSUV at Wrist	3.3 ± 1.9	2.5 ± 1.6	2.9 ± 2.2	1.5 ± 0.1	3.0 ± 2.0	0.306

Data are given as mean ± SD. hSUV, highest maximum standardized uptake value; RA, rheumatoid arthritis; OA, osteoarthritis; Other, other rheumatic diseases; FM, fibromyalgia.

### Diagnostic performance of joint SUV for discerning RA or FM

**Figure 3:**
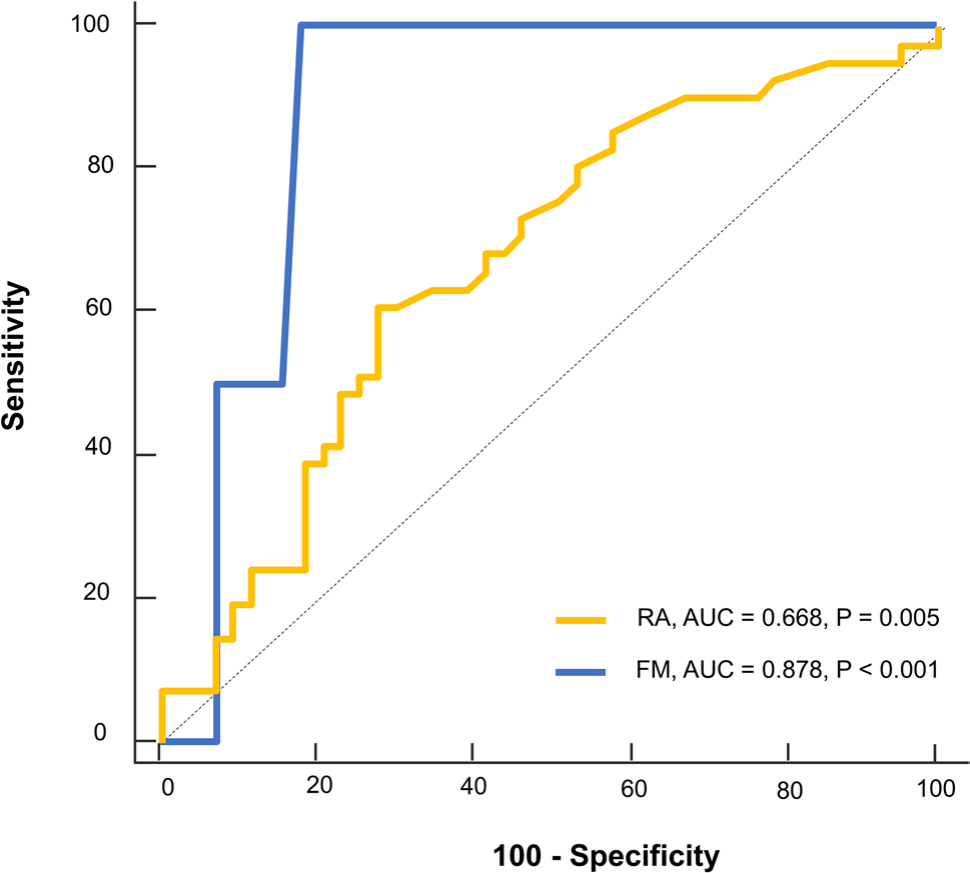
ROC curves illustrating the role of cumulative joint hSUVs in differentiating RA (A) or FM (B) from other rheumatic diseases.

Using summed hSUVs at individual joint groups, we analyzed the best predictive performance of ROC analysis for RA and FM. The cumulative hSUV of the PIP, MCP, and wrist joints showed good predictive performance (AUC, 0.668; *P* = 0.005; Fig. 3). When an SUV of 7.4 was used as an optimal criterion for predicting RA patients, the diagnostic sensitivity and specificity were 61.0 and 72.1, respectively.

**Figure 4:**
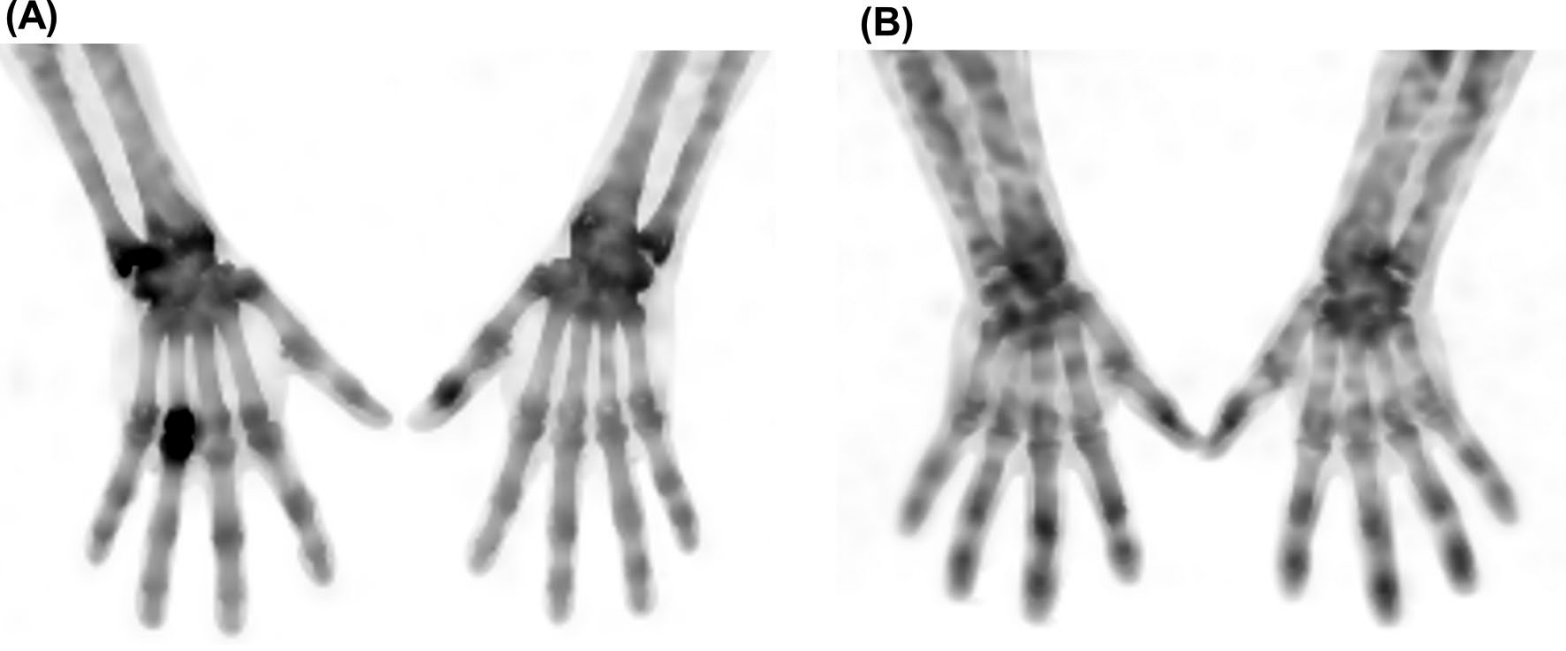
Representative xSPECT images (**A**), (**B**) for differentiating RA from FM using the highest SUVs in the hand and wrist joints. A maximum intensity projection (**A**) image of an 86-year-old female with RA, showing a summed hSUV of 19.6 in the PIP, MCP, and wrist joints. A maximum intensity projection (**B**) image of a 30-year-old female showing a low summed SUV of 5.1 at all joint levels. The patient was diagnosed with fibromyalgia.

The summation of hSUVs at four joint groups had excellent performance for predicting FM (AUC, 0.878; *P* < 0.001; Fig. 3). When an SUV of 5.9 was used as an optimal criterion for predicting FM, the diagnostic sensitivity and specificity were 100.0 and 81.7, respectively. Representative SPECT/CT images of a RA patient with high SUV at the PIP, MCP, and wrist joints, and an FM patient with low SUV at all joints are illustrated in Fig. 4.

## Discussion

In medical imaging, quantitative measurements can potentially overcome the subjective bias of purely visual analysis^12^. Modern methods of iterative SPECT/CT reconstruction enable the quantitative estimation of local tracer uptake and are more cost-effective than positron emission tomography (PET)^13^. In recent studies, lesion SUVs measured via quantitative bone SPECT/CT showed high accuracy for distinguishing malignant conditions from benign lesions^14,15^. It also appears to be useful to objectively evaluate disease severity in arthritic disease in the knee or temporomandibular joints^2,3^. In the present study, the quantitative method using the SUVmax showed the potential for differentiating articular problems from pain perception in rheumatic patients with hand and wrist pain. To our knowledge, this was the first clinical study to evaluate the diagnostic utility of quantitative bone SPECT/CT in patients with hand or wrist pain. Especially, this finding may provide objective information to differentiate soft tissue rheumatism such as FM from inflammatory arthritis including RA and OA.

Pain is a significant component of many rheumatological conditions and is the result of a complex physiological interaction between central and peripheral nervous system signaling. Due to the very large inter-individual differences in these central nervous system factors that influence pain perception, the severity of pain is often not consistent with the degree of tissue inflammation or damage. In this trend, joint SUV measured via quantitative SPECT/CT may objectively and directly represent inflammation and/or damage in peripheral structures, rather than pain perception. In our study, painful hand and wrist sites were significantly associated with relatively high joint SUVs. However, there were also considerable lesions with inconsistency between the presence of pain and the magnitude of the joint SUV. This large overlap between painful and pain-free joint SUVs may suggest that there are various pathophysiological causes for painful hand and wrist joint symptoms besides altered bone and joint activity. Related to this finding, distribution and intensity in joint SUV differed from pain according to specific rheumatic diseases. In particular, RA showed higher joint SUVs and FM lower SUVs, presumably associated with less arthritic activity and more pain sensitization.

FM is one of the most common types of non-articular rheumatic disorders and can be misdiagnosed as other inflammatory arthritis conditions associated with several overlapping features^16,17^. FM may show higher measures of pain intensity and disease status for the same degree of inflammation or structural damage as the central pain processing system^18^. Therefore, to distinguish FM from other inflammatory arthritis conditions, it is more helpful to determine the degree of joint inflammation than to assess pain. Bone scintigraphy is a useful imaging tool to recognize the presence of joint problems. However, visual assessments have interobserver variability in determining the presence of abnormal joint lesions. For example, diffusely increased physiologic uptake without definite focal activity in the small joints of the hand can sometimes be misinterpreted as diffuse joint problems. In comparison, quantitative analysis can provide more objective information for arthritic activity than visual assessments. ROC curve analysis using the cumulative SUV of all joints showed the best performance in differentiating FM from other rheumatic diseases. However, further studies including a large number of subjects are required to elucidate the diagnostic utility of this quantitative parameter for FM due to the small sample size in the present study.

RA and OA are the two most common forms of arthritis involving hand and wrist joints. RA is an autoimmune and inflammatory process involving joints, especially synovial membranes, whereas OA is caused by mechanical wear and tear of the joints. Despite varying pathogenesis, the differentiation of these conditions can be challenging because they often cause similar symptoms, particularly in the early stages. In our study, RA consistently showed higher frequency and intensity of arthritic activity based on SUV measurements compared to OA. This result may be associated with the higher bone turnover caused by greater inflammatory reactions or specific bone loss in RA^19,20^. In addition, the cumulative highest SUV of the PIP, MCP, and wrist joints displayed an AUC of 0.668 in ROC analysis for distinguishing RA from other rheumatic conditions including OA. However, our study also demonstrated considerable overlap in the joint SUV magnitudes between RA and OA. Therefore, the joint SUV may have limited clinical value for evaluating RA and OA. A better strategy using a quantified method for the clinical utility of bone SPECT/CT in distinguishing RA from other diseases including OA in patients with hand and wrist pain should be explored in future studies.

In this study, the radioactive uptake of hand and wrist joints on quantitative SPECT/CT was measured as the SUVmax, which has been widely used in previous studies. In previous studies using bone scintigraphy, the quantification of abnormal joint activity was evaluated by specific patient measurements such as the joint-to-background activity^21^. However, the degree of radiotracer uptake in the reference background region can often be affected by blood flow and the rate of new bone formation^22,23^. Especially, the presence of inflammatory conditions or age-related changes such as osteoporosis may potentially lead to an overestimation or underestimation of joint activity. Therefore, the joint SUVmax may reflect arthritic activity better than patient-specific measurements. Consistent with this, a previous study reported that the joint SUVmax on SPECT/CT had better performance in evaluating temporomandibular joint disease than the relative uptake ratio^3^. However, the threshold of the SUVmax to define abnormal activity has not yet been established in hand and wrist joints. Although the active joint site in our study was categorized using the median SUV in the same joint group (DIP, PIP, MCP, and wrist) in the total population, it is not actually abnormal lesion but site with relatively high uptake in preselected clinical patients. To differentiate abnormal activity from normal physiologic uptake, the normal reference for joint SUV should be investigated in future studies including normal controls.

The major limitations of this study were its retrospective design and a rather modest number of subjects. Our study population was also highly selected by clinicians, which might have influenced the results as selection bias. The SUVmax threshold and performance in hand and wrist joints were derived from patient groups with rheumatic conditions. Future studies with more adequate control patients are required to strengthen our findings and further confirm the potential utility of bone SPECT/CT in the evaluation of patients with hand and wrist pain.

## Conclusion

The joint SUVs in hand and wrist on bone SPECT/CT images exhibits lower magnitude in FM with non-articular pain than in inflammatory arthritis with articular pain. Our preliminary result demonstrated the quantitative bone SPECT/CT might be considered as more objective and standardized technique in assessing joint activity for rheumatic disease patients. In the future, prospective studies with a larger sample size including normal controls should be conducted to confirm the diagnostic efficacy of quantitative bone SPECT/CT for patients with hand and wrist pain.

## Supplementary Information

Supplementary Figure S1.

## Data Availability

All data generated or analyzed in this study are included in this published article.
