# Supplementary material for: Clinical significance of quantitative bone SPECT/CT in the evaluation of hand and wrist pain in patients with rheumatic disease
Source: Sci Rep. 2022 Jan 10;12:327. doi: 10.1038/s41598-021-03874-9 (PMC8748937; doi:10.1038/s41598-021-03874-9)

***<u>Supplementary Material</u>***

# Clinical Significance of Quantitative Bone SPECT/CT in the Evaluation of Hand and Wrist Pain in Patients with Rheumatic Disease

Chae Hong Lim[1†], Hyun-Sook Kim[2†], Kyung-Ann Lee[2], JongSun Kim[2], Soo Bin Park[1]

[1]Department of Nuclear Medicine; Soonchunhyang University College of Medicine, Seoul, Korea

[2]Department of Rheumatology; Soonchunhyang University College of Medicine, Seoul, Korea

† Chae Hong Lim and Hyun-Sook Kim contributed equally to this study as co-first authors

Supplementary Figure

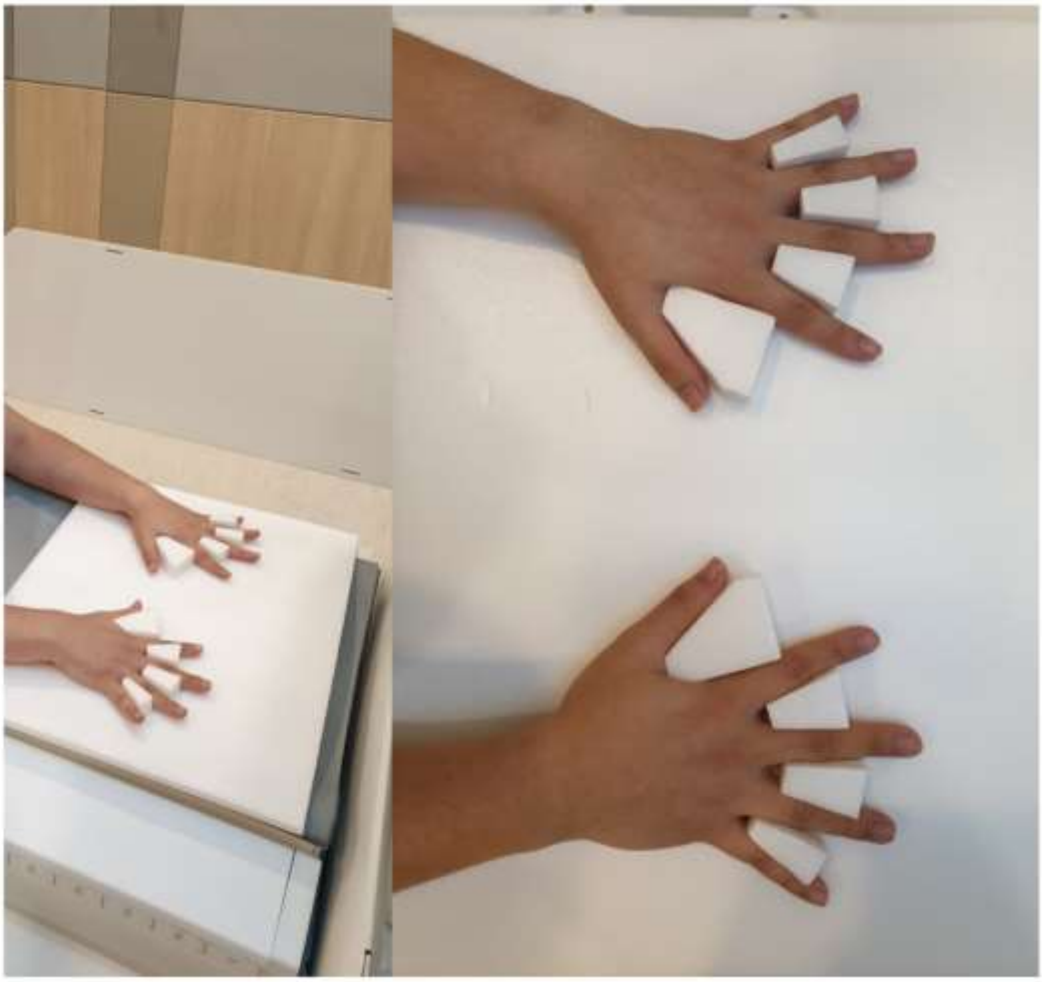

Supplement: Supplementary file 1 — Supplementary Figure S1. [file 41598_2021_3874_MOESM1_ESM.pdf]
